# An Energy-Aware Runtime Management of Multi-Core Sensory Swarms

**DOI:** 10.3390/s17091955

**Published:** 2017-08-24

**Authors:** Sungchan Kim, Hoeseok Yang

**Affiliations:** 1 Division of Computer Science and Engineering, Chonbuk National University, 567 Baekje-daero, deokjin-gu, Jeonju-si, Jeollabuk-do 54896, Korea; s.kim@chonbuk.ac.kr; 2 Department of Electrical and Computer Engineering, Ajou University, 206 Worldcup-ro, Yeongtong-gu, Suwon-si 16499, Korea

**Keywords:** sensory swarm, energy minimization, multi-core processor, dynamic voltage frequency scaling (DVFS), self-adaptation, runtime resource management

## Abstract

In sensory swarms, minimizing energy consumption under performance constraint is one of the key objectives. One possible approach to this problem is to monitor application workload that is subject to change at runtime, and to adjust system configuration adaptively to satisfy the performance goal. As today’s sensory swarms are usually implemented using multi-core processors with adjustable clock frequency, we propose to monitor the CPU workload periodically and adjust the task-to-core allocation or clock frequency in an energy-efficient way in response to the workload variations. In doing so, we present an online heuristic that determines the most energy-efficient adjustment that satisfies the performance requirement. The proposed method is based on a simple yet effective energy model that is built upon performance prediction using IPC (instructions per cycle) measured online and power equation derived empirically. The use of IPC accounts for memory intensities of a given workload, enabling the accurate prediction of execution time. Hence, the model allows us to rapidly and accurately estimate the effect of the two control knobs, clock frequency adjustment and core allocation. The experiments show that the proposed technique delivers considerable energy saving of up to 45%compared to the state-of-the-art multi-core energy management technique.

## 1. Introduction

Future computer systems are expected to be networks of mobile and stationary devices which exchange a huge amount of information between them [1]. At the edge of such distributed computing frameworks, there are low-end embedded systems referred to as *sensory swarms* that deal with the acquisition and pre-processing of data obtained from sensors. As such systems become smarter, it is expected that more computationally intensive applications will be executed on the devices [2,3,4]. In order to handle such heavy computational loads, multi-core processors have now become common in the design of sensory swarms or wireless sensor networks (WSNs) [5,6].

Even though the multi-core processor design significantly enhances the compute capability, this benefit comes at the cost of increased energy consumption. Moreover, since most sensory swarm devices or WSNs are battery-powered, energy minimization with respect to a given performance requirement is usually considered a top priority in the design of sensory swarms. In general, it is impractical to design an application that meets the performance constraint for all possible hardware platforms at design time. Furthermore, as the data collected through sensors affects CPU workload [7], it is crucial to consider such dynamic computational demand in the optimization of sensory swarms. Furthermore, the performance requirements are no longer constant for all operation times in modern sensor swarms or WSNs. As can be seen in context-aware tracking [8] or surveillance [9], the performance goals that the devices have to meet are dynamically changing. Thus, we propose to take a runtime approach as a solution to this challenge. That is, we periodically monitor the runtime performance of a system and reconfigure the system in adaptation to dynamically varying workload. The number of cores that are used for the execution and their clock frequencies are the control knobs used for this reconfiguration procedure.

Hoffmann et al. [10] proposed a light-weight and portable software monitoring runtime, based on which core allocation/scheduling is reconfigured online. Sironi et al. [11] proposed PAFS (Performance-Aware Fair Scheduler), which is a self-adaptive multi-core scheduler customized from the Linux CFS (Completely Fair Scheduler). Sarma et al. [12] also proposed a measurement-based adaptive scheduling policy for multi-core Linux. Al Faruque et al. [13] proposed an adaptive core allocation technique for multi-core NoCs (networks-on-chips) in reducing the network traffic online as a response to various hard-to-predict system scenario changes. Hoffman et al. [14] also proposed to reconfigure software parameters at runtime in order to satisfy performance requirements in varying workload and power budget. Rangan et al. [15] proposed a thread-level runtime power management technique for multi-core systems. Similarly, Yun et al. [16] devised a holistic multi-core reconfiguration technique that deals with power management and core allocation at the same time. Li et al. [17] also proposed the co-optimization of the dynamic voltage frequency scaling (DVFS) with task allocation/scheduling on multi-core systems. However, it is different from the previous ones in that it takes a hybrid approach of static scheduling and dynamic power management.

The proposed technique differs from the existing ones in three respects. First, it is more general and portable. That is, previous approaches mainly limit themselves to only a single control knob such as core allocation/scheduling [10,11,12,13] or clock frequency (and voltage) scaling [15,17], while the proposed technique considers both at the same time. Furthermore, it does not require any modifications of scheduling kernel, unlike [11,12] or application codes on the contrary to [14]. Secondly, we adopt a more practical performance model based on the measurement of IPC (instructions per cycle) that represents the intensity of memory access, thus enabling more accurate reconfiguration. The approach proposed by Yun et al. [16] is similar to ours in adapting two control knobs, but unlike ours its performance model ignores different compute-intensities of applications, resulting in inaccurate reconfigurations. The third difference is in the fact that the existing reconfiguration techniques require accurate performance and energy estimations considering the detailed information of underlying hardware, such as micro-architectural parameters of pipeline structure and multi-level cache hierarchy [18,19]. However, building such accurate models is not always possible for all target systems, making the general applicability of those techniques very poor. On the contrary, we devise a practical and portable performance/energy model which necessitates only instruction count and simple power equations.

Based on the simple yet effective performance-energy model, we propose a runtime management technique of processing cores for a multi-threaded (or multi-tasked) application under performance constraints, aiming at energy minimization. As stated above, two design parameters are considered as control knobs: the number of cores allocated to an application and the clock frequencies thereof. This reconfiguration decision is made periodically; thus, the runtime performance of the target application needs to be periodically measured to capture the current computational demand. In order to avoid complicating the decision, we devised a lightweight and incremental reconfiguration algorithm. We show that the proposed approach achieves considerable energy saving of up to 45% compared to the state-of-the-art presented in [16].

The rest of this paper is organized as follows. In the following section, we show the overall framework of the proposed technique, including the application and architecture models. As we consider multiple control knobs at the same time, we propose a novel system adaptation algorithm in Section 3. Then, in Section 4, we show experimental results with real-life benchmarks, which is followed by concluding remarks.

## 2. Proposed Self-Adaptive Framework

In this section, we describe the proposed self-adaptive sensory swarm design. First, in Section 2.1, we illustrate the task and architecture models assumed in this work. Then, we show how we dynamically and adaptively change the system configuration in response to workload variations in Section 2.2.

### 2.1. System Model

**Hardware:** We consider a hardware platform that has a homogeneous multi-core processor with DVFS enabled. It is assumed that the clock frequencies of main memory and I/O are fixed.

**Application:** Following the multi-frame task [20] (a popularly adopted model in real-time scheduling), we assume that an application consists of a series of execution phases. That is, an application task *T* is defined as a finite vector of execution segments, $E_{i}$, as follows:

(1) $$T=\left[E_{1},E_{2},\ldots ,E_{n}\right].$$

Note that an application is assumed to be instantiated repeatedly, starting from the beginning again when it finishes the execution of its last segment. The time interval between the beginnings of two consecutive instances of *T* is denoted by *epoch*, and an application is given a range of throughput constraints (quality of service (QoS) requirements) as $\left[th_{min},th_{max}\right]$ in epochs/s, within which the reciprocal of the epoch interval time should always be.

Each execution segment is either *sequential* or *parallel*; that is, $type(E_{i})=s$ if $E_{i}$ is a sequential segment, while $type(E_{i})=p$ otherwise. When an execution segment only requires a single core execution, it is referred to as a sequential phase. Otherwise, an execution segment is assumed to have multi-core workload and called parallel phase. This model can be seen as a general fork-join task model, which is equivalent to the state-of-the-art multi-processor programming models [21,22,23,24].

**Figure 1:**
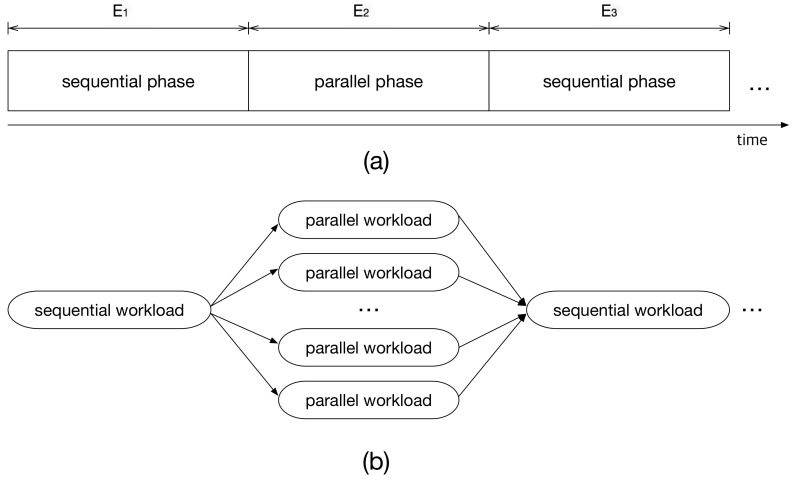
(**a**) A multi-frame task example and (**b**) its fork-join model representation.

Figure 1a shows an example of a multi-frame task, $\left[E_{1},E_{2},E_{3},\ldots \right]$, where $E_{2}$ is a parallel phase. A parallel phase can be executed simultaneously on multiple cores. Thus, this can be modeled as diverged and independent execution paths in the task graph as shown in Figure 1b. That is, if we have multiple processing elements and operating system level supports like multi-threading or multi-tasking, this segment can be executed on more than one cores, resulting in reduced execution time. Note that the degree of parallelism—the number of divergent paths for $E_{2}$ in Figure 1b—is typically larger than the number of cores in the system, and the number of cores to be allocated can change at runtime.

**Execution Time:** The execution time of an execution phase $E_{i}$ consists of CPU execution $E_{i,cpu}$ and memory access time $E_{i,mem}$. That is,

(2) $$E_{i}=E_{i,cpu}+E_{i,mem}.$$

Note that we assume that the performance of memory and I/O buses is fixed at all times. Thus, $E_{i,mem}$ is agnostic to the hardware configuration (though hardware configuration is a general term that refers to any settings in a device, we use this in a limited sense, in which only clock frequency and core assignment are adjustable). On the other hand, the CPU execution time, $E_{i,cpu}$, is dependent upon computing capability dynamically adjusted by the core configurations. Given a hardware configuration tuple (clk,m), where clk and *m* denote the clock frequency of cores and the number of cores allocated when executing $E_{i}$, respectively, the CPU execution time of a sequential phase $E_{i}$ can be approximated as follows:

(3) $$\forall E_{i}\;s.t.\;type\left(E_{i}\right)=s,E_{i,cpu}\left(m,clk\right)\approx (\mathrm{number}\;\mathrm{of}\;\mathrm{cycles}\;\mathrm{taken})\cdot (\mathrm{clock}\;\mathrm{period})=\frac{I_{s}}{\alpha }\cdot \frac{1}{clk}$$

where the actual issue width of a superscalar processor is α and the number of instructions within the segment is $I_{s}$. As shown in Equation (3), the number of assigned cores does not make any difference in the CPU execution time of a sequential phase. It is worth mentioning that $I_{s}$ can be easily measured by means of the integrated hardware performance counter [25].

On the contrary to sequential phases, the CPU execution time of a parallel phase is also affected by the number of cores *m*, as follows:

(4) $$\forall E_{i}\;s.t.\;type\left(E_{i}\right)=p,E_{i,cpu}\left(m,clk\right)\approx \frac{I_{p}\left(m\right)}{\alpha }\cdot \frac{1}{clk}.$$

In the above equation, $I_{p}\left(m\right)$ denotes the number of instructions of *each* of *m* cores in the corresponding phase. The more cores assigned to the application, the fewer instructions executed on each core.

For the brevity of presentation, let us define $E_{s}$ of application *T* as the sum of all sequential phases in *T*. That is, $E_{s}:=\Sigma_{type(E_{i})=s}E_{i}$. Likewise, we will use simplified notations $E_{s,cpu}$, $E_{s,mem}$, $E_{p,cpu}$, $E_{p,mem}$, and $E_{p}$ for the rest of this paper.

### 2.2. Overall Framework

**Figure 2:**
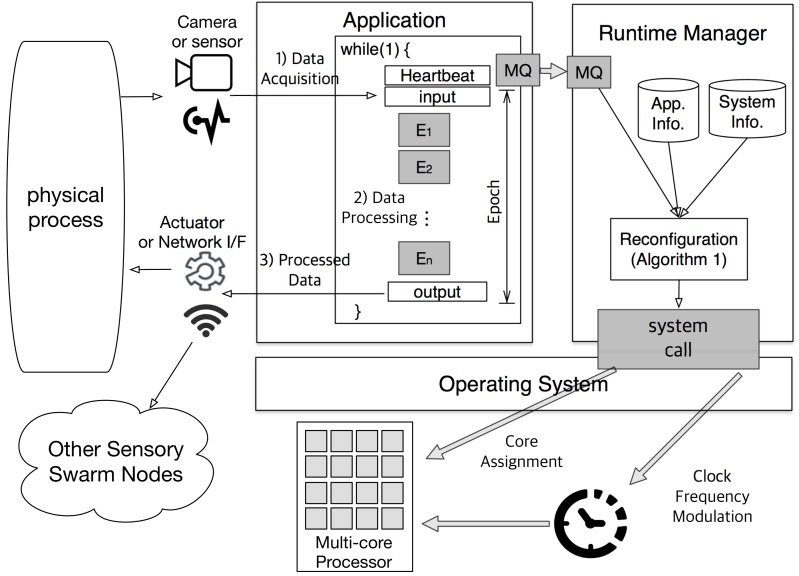
Overall framework of the proposed self-adaptive multi-core sensory swarm node.

Figure 2 illustrates the overall framework of the proposed self-adaptive sensory swarm architecture. The operation of sensory swarms can be modeled as a repetition of the following three steps as shown in the left-hand side of Figure 2: (1) data acquisition from sensors, (2) data processing, and (3) triggering actuators or communications based on the processed data. Among them, we focus on the data processing part, which is the main source of CPU workloads. As described in the previous subsection, the data processing part is implemented as an ever-repeating loop (*while* (1) in the figure) of the multi-frame task segments. At the beginning of each loop execution, the application records a timestamp using the *heartbeat* framework [10]. Then, the interval between two consecutive heartbeats corresponds to *epoch* and the heartbeat rate is calculated as the reciprocal of epoch duration. As stated in the system model, the application is given a heartbeat range, $\left[th_{min},th_{max}\right]$ in epochs/s, as a performance constraint.

Other than the application task (which deals with data processing), there is another task called runtime manager running in the sensory swarm system. Each time the application records the heartbeat, the runtime manager checks whether the given performance condition is satisfied or not, and performs a system reconfiguration if necessary. This reconfiguration procedure is described in detail in the next section. In order to maintain the general applicability of the proposed technique, we use the existing Linux commands or system calls without any modifications for the reconfiguration. To be more specific, we use the *taskset* command for core assignment and the *cpufreq* file for frequency modulation.

## 3. Proposed Self-Adaptive Reconfiguration Policy

Algorithm 1 illustrates how the proposed technique responds to a throughput requirement violation. The adaptation is performed when the heartbeat rate violates the constraint. The optimal adjustment of the control knobs may require an exploration of too many possible cases, leading to substantial runtime computational overhead. Thus, we confine ourselves to incremental adjustments such as adding (or releasing) a core or scaling clock frequency a single step higher (or lower). In case the performance is under the lower bound of the constraint (lines 3–10), either increasing clock frequency or allocating one more core is chosen. On the other hand, if the performance monitored is above the upper bound of the constraints (lines 11–18), either decreasing clock frequency or releasing a core is considered as a response to less computational workload. Note that one of the two control knobs (i.e., clock frequency or core assignment) is to be chosen as a reconfiguration solution each time Algorithm 1 is called. Thus, we need to quantitatively compare them in terms of performance per energy gain in $gain_{f}$ and $gain_{c}$ as shown in lines 4–5. In what follows, we explain how we estimate the effect of each adjustment decision.

**Algorithm 1** Self-adaptive reconfiguration procedure. 1: **while** an application is running **do** 2:    h← heartbeat rate of the current epoch 3:    **if**
$h<th_{min}$
**then** 4:          $gain_{f}\leftarrow EFF_{s,f}+EFF_{p,f}$ 5:          $gain_{c}\leftarrow EFF_{p,c}$ 6:          **if**
$gain_{c}<gain_{f}$
**then** 7:             Scale up clock frequency by a single step 8:          **else** 9:             Allocate one more core unless all cores are busy10:         **end if**11:    **else if**
$h>th_{max}$
**then**12:         $loss_{f}\leftarrow EFF_{s,f}+EFF_{p,f}$13:         $loss_{c}\leftarrow EFF_{p,c}$14:         **if**
$loss_{f}<loss_{c}$
**then**15:            Scale down clock frequency by a single step16:         **else**17:            Release one core unless only one core is busy18:         **end if**19:    **end if**20: **end while**

### 3.1. Predicting Energy Impact of Core Allocation Policy

We use the predicted performance gain per increased energy as an indicator for choosing a suitable adjustment. Similarly, the performance loss per energy saving should also be quantitatively considered in case of slowing down. It is worth mentioning that performance per power or energy consumption is a popular metric to quantify energy efficiency [26,27]. We first examine the effect of frequency scaling. Let $\Delta EN_{s,f}\left(n,clk,clk^{\prime }\right)$ be the increment in energy consumption for the *sequential* phase due to scaling up the clock frequency from clk to $clk^{\prime }$ while preserving the number of assigned cores as *n*.

That is,

(5) $$\Delta EN_{s,f}\left(n,clk,clk^{\prime }\right)=E_{s}\left(n,clk^{\prime }\right)\cdot P\left(clk^{\prime }\right)-E_{s}\left(n,clk\right)\cdot P\left(clk\right),$$

where P(clk) is the power consumption of a core running at clk. The core power consumption model is empirically derived as detailed in the next section.

Similarly, the increment in energy consumption of *parallel* phases due to the frequency scaling $\Delta EN_{p,f}$ can also be formulated as follows:

(6) $$\Delta EN_{p,f}\left(n,clk,clk^{\prime }\right)=n\cdot \left(E_{p}\left(n,clk^{\prime }\right)\cdot P\left(clk^{\prime }\right)-E_{p}\left(n,clk\right)\cdot P\left(clk\right)\right).$$

Note that the parallel phases affect the energy consumption of multiple *n* cores. It is worth mentioning that the proposed energy model is only concerned with the computational workload of the CPU. As the proposed technique does not consider the memory DVFS and the core allocation does not make any significant differences in memory accesses, this modeling is valid enough to tell the *relative* energy consumptions of the two control knobs.

In order to assess the effectiveness of the frequency scaling, we define $EFF_{s,f}$ and $EFF_{p,f}$ as the predicted performance gains per unit energy increase in sequential and parallel phases, respectively. That is,

(7) $$EFF_{s,f}=\frac{E_{s}\left(n,clk\right)-E_{s}\left(n,clk^{\prime }\right)}{\Delta EN_{s,f}\left(n,clk,clk^{\prime }\right)}$$

and

(8) $$EFF_{p,f}=\frac{E_{p}\left(n,clk\right)-E_{p}\left(n,clk^{\prime }\right)}{\Delta EN_{p,f}\left(n,clk,clk^{\prime }\right)}.$$

Now, we have an indicator of the effectiveness of the frequency scaling, $gain_{f}$, as the sum of Equations (7) and (8).

We basically follow the same principle in quantifying the effectiveness of the core assignment. The only difference is that we do not need to use more than a core in sequential phases; i.e., $\Delta EN_{s,c}=0$. On the other hand, it does increase the energy consumption in parallel phases. The energy increment in parallel phases $\Delta EN_{p,c}$ is formulated as follows when the number of allocated cores changes from *n* to $n^{\prime }$ such that $n^{\prime }>n$:

(9) $$\Delta EN_{p,c}\left(n,n^{\prime },clk\right)=P\left(clk\right)\cdot \left(n^{\prime }\cdot E_{p}\left(n^{\prime },clk\right)-n\cdot E_{p}\left(n,clk\right)\right).$$

Then, the effectiveness of adding more cores on parallel phases is

(10) $$EFF_{p,c}=\frac{E_{p}\left(n,clk\right)-E_{p}\left(n^{\prime },clk\right)}{\Delta EN_{p,c}\left(n,n^{\prime },clk\right)}.$$

Again, adjusting core allocation does not affect the sequential phase; i.e., $EFF_{s,c}=0$. Thus, $gain_{c}=EFF_{EFF_{p,c}}$. Now that we have both $gain_{f}$ and $gain_{c}$, we can tell which one is the more suitable reconfiguration policy. As shown in lines 6–10 of Algorithm 1, the one which has a bigger gain value will be chosen as a reconfiguration policy for the next epoch.

The same principle applies to the case of reconfiguring systems to run slower (lines 11–18). In such cases, EFFs can be understood as performance loss per energy saving (lines 12–13), and the option with smaller value is adopted as a next configuration. Namely, when decreasing the clock and releasing a core have the same energy savings, the one with less execution time increments is chosen. On the other hand, when they tie in the execution time increments, the system is adjusted to the one with larger energy savings.

While this study focuses on the frequency scaling and core assignment, the proposed framework is not limited to any specific control knobs. In other words, the proposed technique can be extended to consider other control knobs, once the performance and energy of the target system can be properly modeled with them. For instance, Equations (5) and (6) can be extended to consider voltage scaling with a modification of function P(·). Likewise, as another example one may consider heterogeneous multi-cores in the reconfiguration by enhancing Equations (3) and (4).

## 4. Experiments

**Hardware platform and configuration:** We conducted experiments on the Tegra-K1 system that consists of a quad-core ARM Cortex-A15 processor [28]. The clock frequency of the processor scales from 204 MHz to 2.3 GHz in steps of 100 MHz.

**Benchmarks:** We took an image processing application—*Heart-Wall*—from the Rodinia benchmark suite [29] and a particle filter-based *Object tracking* application as benchmarks, both of which exhibit per-frame workload variations. Image processing and object tracking applications are among commonly used applications for high-end WSNs or sensory swarms [30,31]. Note that the workload characteristics involved in the benchmarks are different. In particular, *Heart-Wall* is compute-intensive with relatively consistent processor utilization, while memory access behavior of *Object tracking* is quite nondeterministic due to the stochastic nature of the particle filter. We implemented the proposed technique described in Algorithm 1 by augmenting the heartbeat APIs [10] into the beginning of the outer-most loop in the benchmarks to monitor the runtime performance and to perform the required adaptation.

The superscalar issue width α in Equation (3) is set to two considering the average behavior of Cortex A-15. We measured instruction counts, $I_{s}$ and $I_{p}\left(\cdot \right)$, using a built-in hardware performance counter [25], which are non-intrusive and exhibit negligible performance overhead. We empirically established the power model of a single core P(·) in Equations (5), (6), and (9). Concretely, we ran a compute-intensive workload *Sample_PI* repeatedly, in which a processor is known to cause negligibly little memory accesses [32]. Gradually increasing the core frequency, we measured the system power consumption, then built a simple prediction model of core power consumption using linear regression.

We compare the proposed approach with a state-of-the-art work [16] where the adaptation is done by exhaustively searching the energy-optimal configurations combining frequency scaling and core allocation. In [16], the design space is predetermined to reduce the computation overhead taken for search, using the notation of *distance*. In particular, the distance of two configurations is defined as total disparity in the control knob adjustments, core allocation, and frequency scaling. Taking this approach, we consider all configurations with less than distance of 8 from the current configuration during the adaptation. This method is referred to as *Exhaustive* hereafter. We also take a default Linux scheduler with the *high-performance* governor as *Baseline*.

**Figure 3:**
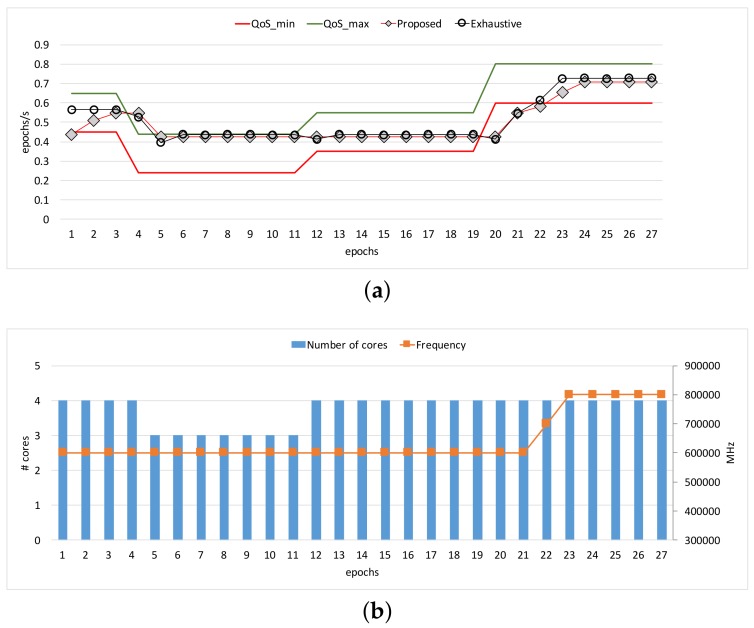

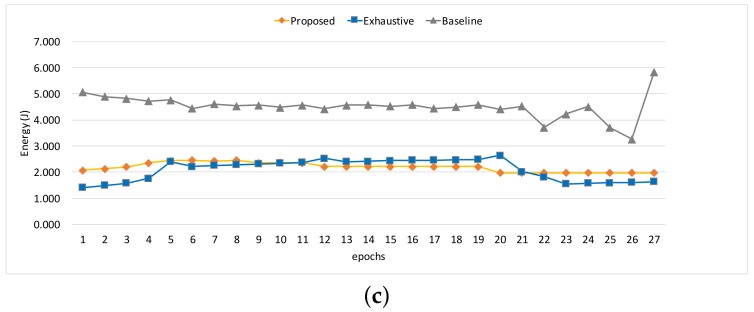
Comparisons of our proposed approach with the *Baseline* and *Exhaustive* approaches under smooth workload (*Heart-Wall*): (**a**) performance, (**b**) hardware configurations, and (**c**) energy consumptions.

**Figure 4:**
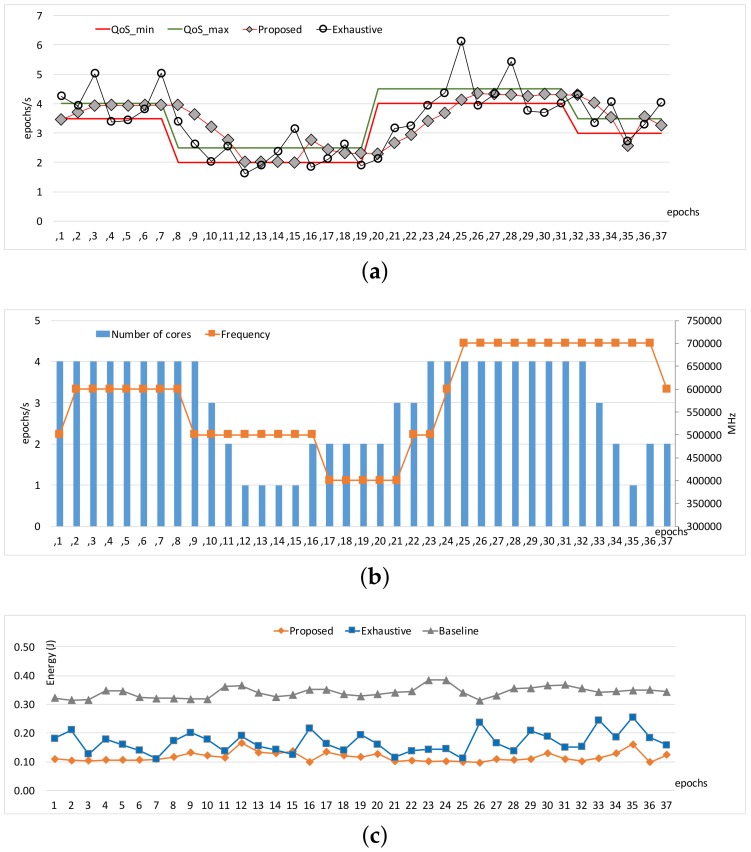
Comparisons of our proposed approach with the *Baseline* and emphExhaustive approaches under heavily varying workload (*Object-tracking*): (**a**) performance, (**b**) hardware configurations, and (**c**) energy consumptions.

Figure 3 and Figure 4 show the comparisons of the three approaches in terms of workload adaptation and corresponding energy consumption over time. The throughput constraints were set to vary as depicted with the dotted lines in Figure 3a and Figure 4a. We also provide the configuration of the two control knobs as counterparts at the same epochs for each of the benchmarks in Figure 3b and Figure 4b, respectively. Note that we exclude a warm-up stage of the first few epochs until which the performance exhibited with our approach reached the lower bound of the constraints because the configurations were initially set to the lowest possible compute capability. The performance traces of *Baseline* are omitted here because it is not designed to adapt to the given constraints; only its energy consumptions are shown for comparison in Figure 3c and Figure 4c. When the memory access behavior is stable as shown in *Heart-Wall*, the proposed approach and *Exhaustive* perform similarly in throughput.

The reason why *Exhaustive* performs relatively well partly in Figure 3a is largely due to the fact that the stable and compute-intensive workload of *Heart-Wall* is favorable for the performance model in [16], which unlike ours ignores the impact of memory-intensity on performance. However, in the cases where the memory-intensity of workload severely changes in a nondeterministic way (as is in *Object tracking*), the performance model of *Exhaustive* becomes inaccurate, and thus the adaptation tends to oscillate as shown in Figure 4a. On the other hand, our approach adapts to the workload variations smoothly compared to *Exhaustive*. In turn, such better adaptivity leads to a higher energy efficiency, as is quantified in Figure 4b.

We observe that the different patterns of resource management appear according to workload characteristics, as shown in Figure 3b and Figure 4b. The proposed technique adapts for *Heart-Wall* by primarily changing core allocation over epochs. Since *Heart-Wall* has a compute-intensive workload, it is advantageous to use more cores while keeping clock frequency in terms of energy efficiency as demonstrated by our approach. As a result, the gap between the energy consumptions with the two approaches is marginal as shown in Figure 3c. On the other hand, much complicated behavior of the adaptation appears in the case of *Object tracking*. Both control knobs are active in use over epochs, meaning that workload exhibited in the benchmark has much more memory-centric epochs than those of *Heart-Wall*, requiring careful reconfiguration in the consideration of memory-intensiveness of the workload. Consequently, Figure 3c shows that—unlike the case of *Heart-Wall*—our approach outperforms *Exhaustive* in terms of energy by large margin.

Note that there is still room for further improvement; due to the incremental nature of our approach, there is the potential for resource over-provisioning or constraint violation with steep change in the constraint as shown in epochs 7 and 19 of Figure 4a. More aggressive adjustment could alleviate such drawbacks, which is left as future work.

**Figure 5:**
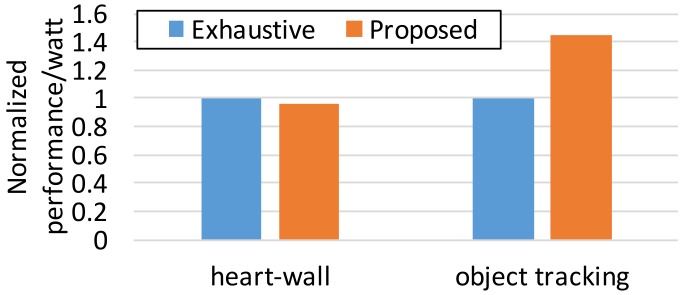
Energy efficiency of two approaches in performance per watt.

Figure 5 shows the energy efficiency of the proposed approach and *Exhaustive* in terms of performance per watt. We sum up the achieved throughput of each epoch and divide it by the accumulated power consumption over epochs. Note that we take the maximum of the constraints as the throughput of an epoch if it actually surpasses the constraints in order to avoid exaggerating the result from the proposed technique. As discussed, *Exhaustive* performs sightly better for the stable and compute-intensive workload, *Heart-Wall*; in spite of its expensive computational cost, *Exhaustive* is just 4.5% better in energy savings, as shown in Figure 5. Note that *Exhaustive* should endure more than 5% of CPU utilization for exploring an optimal configuration candidates [16]. However, our approach is robust in more realistic scenarios, nondeterministic memory-intensity, which leads to the energy saving of 45% compared to *Exhaustive*. In terms of CPU utilization overhead, by limiting the search space of the reconfiguration candidates in Algorithm 1, the CPU utilization overhead is always negligible. This reveals that the accurate modeling of system performance is a key to effective and efficient runtime resource adaptation.

## 5. Conclusions

We presented a runtime management technique for DVFS-enabled multi-core sensory swarms, aiming at minimizing energy consumption under performance constraints. In order to adjust compute capability in response to the dynamically varying workload of an application, the proposed technique considers the runtime adjustment of two control knobs: task-to-core allocation and clock frequency scaling. In order to make an accurate and effective decision, we devised a set of simple performance and energy models for each of the adjustment options. The experimental results showed the considerable energy savings of the proposed technique by up to 45% over the state-of-the-art work. In particular, it was proven to be further effective when the application had a highly varying memory-intensity behavior, which is a realistic and challenging case in real-life sensory swarm systems.

## References

[1] Rabaey J.M. The Swarm at the Edge of the Cloud—A New Perspective on Wireless. Proceedings of the 2011 Symposium on VLSI Circuits (VLSIC).

[2] Martinez-Julia P., Garcia E.T., Murillo J.O., Skarmeta A.F. Evaluating Video Streaming in Network Architectures for the Internet of Things. Proceedings of the 2013 Seventh International Conference on Innovative Mobile and Internet Services in Ubiquitous Computing (IMIS).

[3] Pereira R., Pereira E.G. Video Streaming Considerations for Internet of Things. Proceedings of the 2014 International Conference on Future Internet of Things and Cloud (FiCloud).

[4] Lee J.H., Jang K.S., Kim B.G., Jeong S., Choi J.S. (2015). Fast Video Encoding Algorithm for the Internet of Things Environment Based on High Efficiency Video Coding. Int. J. Distrib. Sens. Netw..

[5] Pang Z. (2013). Technologies and Architectures of the Internet-of-Things (IoT) for Health and Well-being. Ph.D. Thesis.

[6] Wootton C. (2016). Samsung ARTIK Reference: The Definitive Developers Guide.

[7] Marculescu R., Bogdan P. (2011). Cyberphysical systems: Workload modeling and design optimization. IEEE Des. Test Comput..

[8] Gao J., Ling H., Blasch E., Pham K., Wang Z., Chen G. (2013). Pattern of Life from WAMI Objects Tracking based on Context-Aware Tracking and Information Network Models. Proc. SPIE.

[9] Fradi H., Dugelay J.L. (2015). Towards Crowd Density-Aware Video Surveillance Applications. Inf. Fusion.

[10] Hoffmann H., Eastep J., Santambrogio M.D., Miller J.E., Agarwal A. (2010). Application Heartbeats for Software Performance and Health. ACM Sigplan Not..

[11] Sironi F., Bartolini D.B., Campanoni S., Cancare F., Hoffmann H., Sciuto D., Santambrogio M.D. (2012). Metronome: Operating System Level Performance Management Via Self-Adaptive Computing. Proceedings of the 49th Annual Design Automation Conference.

[12] Sarma S., Muck T., Bathen L.A., Dutt N., Nicolau A. SmartBalance: A Sensing-Driven Linux Load Balancer for Energy Efficiency of Heterogeneous MPSoCs. Proceedings of the 2015 52nd ACM/EDAC/IEEE Design Automation Conference (DAC).

[13] Al Faruque M.A., Rudolf K., Jórg H. ADAM: Run-Time Agent-Based Distributed Application Mapping for on-Chip Communication. Proceedings of the 2008 45th ACM/IEEE Design Automation Conference.

[14] Hoffmann H., Sidiroglou S., Carbin M., Misailovic S., Agarwal A., Rinard M. (2011). Dynamic Knobs for Responsive Power-Aware Computing. ACM Sigplan Not..

[15] Rangan K.K., Wei G.Y., Brooks D. (2009). Thread Motion: Fine-Grained Power Management for Multi-Core Systems. ACM SIGARCH Computer Architecture News.

[16] Yun J., Park J., Baek W. (2015). Hars: A Heterogeneity-Aware Runtime System for Self-Adaptive Multithreaded Applications. Proceedings of the 52nd Annual Design Automation Conference.

[17] Li X., Xie N., Tian X. (2017). Dynamic Voltage-Frequency and Workload Joint Scaling Power Management for Energy Harvesting Multi-Core WSN Node SoC. Sensors.

[18] Lukefahr A., Padmanabha S., Das R., Sleiman F.M., Dreslinski R., Wenisch T.F., Mahlke S. Composite cores: Pushing Heterogeneity into a Core. Proceedings of the 2012 45th Annual IEEE/ACM International Symposium on Microarchitecture.

[19] Pricopi M., Muthukaruppan T.S., Venkataramani V., Mitra T., Vishin S. Power-Performance Modeling on Asymmetric Multi-Cores. Proceedings of the 2013 International Conference on Compilers, Architecture and Synthesis for Embedded Systems (CASES).

[20] Mok A.K., Chen D. (1997). A Multiframe Model for Real-Time Tasks. IEEE Trans. Softw. Eng..

[21] Lea D. (2000). A Java Fork/Join Framework. Proceedings of the ACM 2000 Conference on Java Grande.

[22] Dagum L., Menon R. (1998). OpenMP: An Industry Standard API for Shared-Memory Programming. IEEE Comput. Sci. Eng..

[23] CUDA C Programming Guide. http://docs.nvidia.com/cuda/cuda-c-programming-guide.

[24] Khronos Opencl Working Group The Opencl Specification.

[25] Weaver V.M. Linux Perf_Event Features and Overhead. FastPath 2013: Proceedings of the 2nd InternationalWorkshop on Performance Analysis ofWorkload Optimized Systems.

[26] Rodrigues R., Annamalai A., Koren I., Kundu S., Khan O. Performance Per Watt Benefits of Dynamic Core Morphing in Asymmetric Multicores. Proceedings of the 2011 International Conference on Parallel Architectures and Compilation Techniques (PACT).

[27] Kumar V., Fedorova A. (2009). Towards Better Performance per Watt in Virtual Environments on Asymmetric Single-Isa Multi-Core Systems. ACM SIGOPS Oper. Syst. Rev..

[28] (2015). Tegra K1. http://www.nvidia.com/object/tegra-k1-processor.html.

[29] Che S., Boyer M., Meng J., Tarjan D., Sheaffer J.W., Lee S.H., Skadron K. Rodinia: A Benchmark Suite for Heterogeneous Computing. Proceedings of the 2009 IEEE International Symposium on Workload Characterization (IISWC).

[30] Paek J., Hicks J., Coe S., Govindan R. (2014). Image-Based Environmental Monitoring Sensor Application Using an Embedded Wireless Sensor Network. Sensors.

[31] Arora A., Dutta P., Bapat S., Kulathumani V., Zhang H., Naik V., Mittal V., Cao H., Demirbas M., Gouda M. (2004). A Line in The Sand: A Wireless Sensor Network for Target Detection, Classification, and Tracking. Comput. Netw..

[32] Zhao X., Yin J., Chen Z., He S. Workload Classification Model for Specializing Virtual Machine Operating System. Proceedings of the 2013 IEEE Sixth International Conference on Cloud Computing (CLOUD).

